# *Clostridium difficile* Enterocolitis and Reactive Arthritis: A Case Report and Review of the Literature

**DOI:** 10.1155/2016/1591753

**Published:** 2016-04-14

**Authors:** Michela Cappella, Fabrizio Pugliese, Andrea Zucchini, Federico Marchetti

**Affiliations:** Department of Pediatrics, Santa Maria delle Croci Hospital, 48121 Ravenna, Italy

## Abstract

Reactive arthritis is a rare complication of* Clostridium difficile *enterocolitis, especially in children. We review the 6 pediatric cases published in the English and non-English literature and discuss their clinical presentation, outcome, treatment, and pathophysiology. We also report the seventh case of* Clostridium difficile *reactive arthritis in a 6-year-old boy who was treated with amoxicillin-clavulanate for 10 days because of an upper respiratory infection. After the antibiotic course, the child developed at the same time diarrhea with positive stool culture for* Clostridium difficile* and an asymmetric polyarthritis. Nonsteroidal anti-inflammatory drugs and metronidazole completely resolved the pain, joint swelling, and diarrhea. After twelve months of follow-up there has been no recurrence. This report confirms the self-limiting course of* Clostridium difficile* reactive arthritis.* Clostridium difficile* testing in children with gastrointestinal symptoms and acute onset of joint pain should be always considered.

## 1. Introduction

Reactive arthritis (ReA) is an aseptic acute inflammatory arthritis which develops after an extra-articular infection triggered by arthritogenic bacteria, more frequently from* Salmonella*,* Shigella*,* Yersinia,* and* Campylobacter*. It is often associated with the human leucocyte antigen- (HLA-) B27 and usually considered in adult classification as a subtype of spondyloarthropathies. The criteria currently used to diagnose ReA are those of the 1995 Berlin Third International Workshop: (1) the presence of a predominantly lower limb asymmetrical oligoarthritis and (2) clinical laboratory evidence of a preceding infection [1].

A particular type of ReA has been described in connection with* Clostridium difficile* (CD) colitis. Although* Clostridium difficile* ReA is largely described in adults, there are few pediatric case reports probably due to CD asymptomatic colonization in newborns and infants. However, the incidence of CD among hospitalized children has increased in the last few years [2]. To the best of our knowledge, only six cases have been reported in the pediatric population [3–7]. We report the seventh pediatric case of ReA following CD enterocolitis.

## 2. Case Report

A 6-year-old boy was admitted to our department because of pain in the right knee and both ankles started five days before admission. Three weeks earlier he had completed a 10-day course of oral amoxicillin-clavulanate. Off antibiotics, he developed watery diarrhea and abdominal pains.

On admission the child presented with a profuse diarrhea; his left knee and both ankles were swollen, warm, and painful with significant limitation of motion. He could neither stand nor walk because of severe pain. All his other joints were normal. He had no fever. The remaining general examination was unremarkable; in particular he had no rash, mucositis, pharyngitis, urethritis, or conjunctivitis. Initial investigations showed a normal white cells count with a mild increase of C-reactive protein (39 mg/L, normal range 0–5 mg/L) and of the erythrocyte sedimentation rate (30 mm/h; normal range < 20 mm/h); electrolytes were normal. Haemoglobin concentration, platelet count, and fibrinogen were all in the normal range, as antistreptolysin-O (ASO) level (90 KUI/L; normal value < 200 KUI/L).

Three days later the arthritis slowly resolved spontaneously in the left knee and ankles but appeared in the right shoulder and homolateral hip with a migratory pattern. This polyarthritis was accompanied, at that time, with unremitting and not elevated fever (38°C at the maximum). Ultrasonography showed a mild effusion in the right knee, ankles, and right hip joint. X-rays were normal. Both synovial fluid and blood cultures were negative. Tests for immunoglobulin and complement were normal. Autoantibodies (e.g., rheumatoid factor and antinuclear antibodies) were absent and HLA-B27 antigen was negative. Eyes did not show any sign of iridocyclitis on the slit lamp. A cardiac evaluation, including echocardiogram, ruled out any evidence of carditis. Serological tests for arthritis related infections (such as group A* Streptococcus, Borrelia, Cytomegalovirus, *Epstein-Barr-Virus, andParvovirus B19) were also negative.

Our patient's diarrhea, which started following antibiotics, gradually resolved during his hospital stay. Stool cultures for enteric pathogens (such as* Salmonella*,* Shigella*,* Yersinia*,* Campylobacter*, and common viral agent) were negative but CD and CD toxin A and toxin B were detected by ELISA technique. Fecal calprotectin was not increased.

Given positive stool cultures for CD and the polyarthritis, our patient was started on oral naproxen (15 mg/kg for three times a day) for three weeks and metronidazole therapy (7.5 mg/kg every eight hours) for ten days.

Over that period, diarrhea and the polyarthritis rapidly improved. After three weeks of naproxen therapy his symptoms completely resolved. Joint pain abated, the mobility recuperated, and no other joint involvement was noted. Inflammatory parameters turn out to be negative, within seven days after starting treatment. Stool culture after 4 weeks was negative. Fecal calprotectin remained negative.

Our little patient was clinically followed up for 12 months. By then he had completely recovered.

## 3. Discussion

ReA is a nonseptic acute inflammatory arthritis occurring after an intercurrent infection in which the causative organism is outside from the joint. The most frequent causes of ReA are intestinal and genital bacterial infections. ReA is typically characterized by asymmetrical oligo or polyarthritis predominantly in the lower limbs large joints (knee, ankle, and hip) [8, 9]. Symptoms of ReA present themselves approximately from 2 to 4 weeks following infection. The classic triad of arthritis, urethritis, and conjunctivitis, known as Reiter syndrome, is relatively uncommon and the term has been frequently used in the pediatric rheumatology literature in the past [9].

Several patients present a migratory pattern of polyarticular arthritis, but a few have monoarticular or oligoarticular arthritis. Acute arthritis is characterized by severe pain and sometimes erythema over the affected joints but some children may show only light or moderate joint pain and swelling. Enthesitis or tenosynovitis may occur alone or with arthritis. Extra-articular symptoms included systemic signs such as fever, weight loss, fatigue, and skin, eyes, and mucous membranes disease (erythema nodosum, urethritis, conjunctivitis, iridocyclitis, or acute anterior uveitis) [2]. There is a strong association with HLA-B27 antigen and the frequency and severity of joint pain after intestinal or genitourinary infections are higher in HLA-B27 positive patients [9].

ReA can occur at any age, most frequently in young children after enteric infections (*Salmonella*,* Shigella*,* Yersinia enterocolitica*, and* Campylobacter jejuni*), pharyngitis caused by group A* Streptococcus*, or more rarely a genitourinary tract infection (*Chlamydia trachomatis*).

CD is Gram-positive bacillus, spore-forming, obligate anaerobic bacteria and it is the most common cause of antimicrobial-associated diarrhea and colitis after a period of antibiotic therapy [2]. The major risk factor for CD infection in childhood is antibiotic exposure, especially to cephalosporins, broad-spectrum penicillins including amoxicillin, clindamycin, and fluoroquinolones [10]. Infection with toxin-producing strains of CD generally leads to a spectrum of disease ranging from mild, self-limited diarrhea to explosive, watery diarrhea with occult blood or mucous to pseudomembranous colitis.

CD infections are generally more severe in certain populations, including patients receiving chemotherapy or with chronic gastrointestinal diseases [2]. According to the review by Jacobs et al. [10], 35 cases of ReA following CD enterocolitis were documented in adults. The characteristics of CD-associated ReA appeared to be similar both in adults and in children. For the diagnosis of ReaA following CD enterocolitis the criteria established by Putterman and Rubinow [11] may be applied to children. These criteria include the following:

1. Appearance of arthritis together with or following the onset of diarrhea and/or colitis.
2. Diarrhea appearing some time after a course of systemic antimicrobial therapy.
3. Microbiologic proof of CD involvement (either positive stool culture or assay for toxin).
4. No reasonable alternative diagnosis for arthritis or diarrhea (i.e., no other identified infectious agent).

In order to perform a diagnosis of ReA associated with CD enterocolitis, a history of diarrhea after a course of antibiotic or the exclusion of other causes of gastroenteritis or noninfectious arthritis is essential.

Our case fulfills the above criteria by Putterman and Rubinow [11]. Microbiologic confirmation was given by the positive stool culture and CD toxin evidence, together with the absence of other enterobacterial infections.

In addition, antibiotic therapy was previously administered in our patient. There was no evidence of antecedent group A streptococcal infection (ASO title was not raised) and our case did not meet the modified Jones criteria, therefore excluding the diagnosis of acute rheumatic fever or poststreptococcal reactive arthritis. An inflammatory bowel disease (IBD) was considered as it is frequently associated with CD colonization but the dosage of fecal calprotectin, which has a high negative predictive value for IBD, was negative [12]. Moreover, the gradual improvement of colitis allowed the exclusion of IBD. Postinfective arthritis was ruled out because of the negative serological investigation and the polyarticular involvement with migratory patterns.

A literature search of MEDLINE, Google Scholar, and Cochrane Reviews computerized databases using the keywords “*Clostridium difficile*”, “arthritis”, and “child” to identify all papers reporting CD enterocolitis-associated reactive arthritis in childhood was performed. In addition, the search was extended to all relevant articles in the reference lists. No year or language restriction was applied to our search. Two reviewers performed a first stage selection of the total identified studies based on the following inclusion and exclusion criteria (using the abstract or the full text article when required).

All published papers were included in the present review only if they met the following inclusion criteria: (1) appearance of acute arthritis and/or tenosynovitis between 4 weeks before and 12 weeks after the onset of diarrhea and/or colitis; (2) diarrhea appearing some time after a course of systemic antimicrobial therapy; (3) microbiologic proof of CD involvement (either positive stool culture or assay for toxin); (4) no reasonable alternative diagnosis for arthritis or diarrhea (i.e., no other identified infectious agent); (5) negative synovial fluid cultures (if obtained); and (6) age of patient between 1 and 18 years.

Six reports (5 papers) [3–7] met our review's inclusion criteria and their full texts were analysed.  Table 1 shows details regarding these reports on CD ReA in childhood. Cron and Gordon [3] described the first case in 1997 in the USA, and the second one was from the USA as well [4]; thereafter three children in France [5, 6] and one child in the UK [7] were diagnosed with CD ReA. The median age was 6.5 years (range 2.5–11) and there was no age difference between males and females. Arthritis was most often migratory and polyarticular, involving large joints in all cases. CD stool culture was positive in all of them. Only the cases reported by Löffler et al. [5] illustrated that CD-associated colitis was not preceded by antibiotic course but it may be explained by a transient colonization of toxicogenic CD. The duration of the arthritis may be variable. Usually the arthritis resolved from one to four weeks with the use of nonsteroidal anti-inflammatory drugs (NSAIDs). Only in the one case described by Löffler et al. [5] symptoms may persist for more than one year.

In agreement with the other reports, our case confirms the good outcome of ReA following CD infection with a self-limiting course (3 weeks). Only one case documented a child who developed an acute abdomen and underwent a laparotomy for presumed peritonitis [7].

The treatment of ReA following CD infection included the discontinuation of antimicrobial agents and the use of oral NSAIDs coupled with metronidazole therapy for 7–10 days in children and adolescents with mild-to-moderate colitis or vancomycin in more severe diseases. All of previous cases were treated with vancomycin or metronidazole; only the case described by Finger and Neubauer [4] was resolved with only ibuprofen.

## 4. Conclusion

The CD infection should be suspected in children presenting with an acute inflammatory arthritis following an episode of diarrhea especially when stool culture for common enteric bacterial infection turns out to be negative and the patient has received antibiotic therapy before the onset of diarrhea. Therefore, we suggest performing tests for CD toxin in children with these characteristics because CD infection associated with ReA can be unrecognized.

## Figures and Tables

**Table 1:** Case reports in the literature on *Clostridium difficile* enterocolitis-associated reactive arthritis in children.

Authors/articles	Cron and Gordon 1997 [3]	Löffler et al. 2004 [5]		Durand and Miller [7]	Finger and Neubauer 1997 [4]	Dacheux et al. 2012 [6]
*Age*	*2,5*	*11*	*6*	*10*	*3*	*7*
Sex	M	M	F	F	M	M
Previous antibiotic therapy	Amoxicillin	Lincomycin	ND	Erythromycin and penicillin V	Cefixime	Amoxicillin-clavulanate
Joints involved	Left knee and shoulder	Left and right ankles; right knee; both hips; both elbows; left wrist	Both knees, both elbows, and both hips	Left hips	Right hip, shoulder, and knee	Twelve joints
Symptoms associated		Skin nodules		Aseptic peritonitis		
Treatment	Vancomycin	Vancomycin	Vancomycin	Metronidazole	Ibuprofen	Metronidazole
Disease's duration (days)	25	588	14	7	7	7

## Abbreviations

ReA: Reactive arthritis
CD: * Clostridium difficile*
NSAIDs: Nonsteroidal anti-inflammatory drugs.

## References

[1] Kingsley G., Sieper J. (1996). Third international workshop on reactive arthritis. An overview. *Annals of the Rheumatic Diseases*.

[2] Schutze G. E., Willoughby R. E., Committee on Infectious Diseases, American Academy of Pediatrics (2013). Clostridium difficile infection in infants and children. *Pediatrics*.

[3] Cron R. Q., Gordon P. V. (1997). Reactive arthritis to Clostridium difficile in a child. *Western Journal of Medicine*.

[4] Finger D. R., Neubauer J. V. (1997). Reactive arthritis following *Clostridium difficile* colitis in a 3-year-old patient. *Journal of Clinical Rheumatology*.

[5] Löffler H. A., Pron B., Mouy R., Wulffraat N. M., Prieur A.-M. (2004). *Clostridium difficile*-associated reactive arthritis in two children. *Joint Bone Spine*.

[6] Dacheux C., Pruvost I., Herbaux B., Nectoux E. (2012). Clostridium difficile reactive arthritis in a 7-year-old child. *Archives de Pediatrie*.

[7] Durand C. L., Miller P. F. (2009). Severe clostridium difficile colitis and reactive arthritis in a ten-year-old child. *Pediatric Infectious Disease Journal*.

[8] Burgos-Vargas R., Vazquez-Mellado J., Cassidy J. T., Petty R. E. (2010). Reactive arthritis. *Textbook of Pediatric Rheumatology*.

[9] Wiström J., Norrby S. R., Myhre E. B. (2001). Frequency of antibiotic-associated diarrhoea in 2462 antibiotic-treated hospitalized patients: a prospective study. *Journal of Antimicrobial Chemotherapy*.

[10] Jacobs A., Barnard K., Fishel R., Gradon J. D. (2001). Extracolonic manifestations of *Clostridium difficile* infections: Presentation of 2 cases and review of the literature. *Medicine*.

[11] Putterman C., Rubinow A. (1993). Reactive arthritis associated with *Clostridium difficile* pseudomembranous colitis. *Seminars in Arthritis and Rheumatism*.

[12] Ananthakrishnan A. N., Issa M., Binion D. G. (2009). *Clostridium difficile* and inflammatory bowel disease. *Gastroenterology Clinics of North America*.

